# Left Coronary Artery Circumflex Branch Arising From Main Stem of Pulmonary Artery: An Uncommon Anatomical Variation

**DOI:** 10.7759/cureus.15751

**Published:** 2021-06-18

**Authors:** Aikaterini Giannakopoulou, Dimosthenis Chrysikos, Eleftherios Spartalis, Vasileios Protogerou, Theodore Troupis

**Affiliations:** 1 Department of Anatomy, School of Medicine, National and Kapodistrian University of Athens, Athens, GRC; 2 Propaedeutic Surgery, Laiko Hospital, School of Medicine, National and Kapodistrian University of Athens, Athens, GRC; 3 Department of Anatomy, National and Kapodistrian University of Athens, Athens, GRC

**Keywords:** coronary arteries, anatomical, variation, circumflex, pulmonary

## Abstract

The left circumflex coronary artery anatomy is considered highly variable. Herein, we present a case of a 9-year-old male child with a remarkable medical history of a spontaneously closed interventricular septal defect, without residual regurgitation, who was referred for cardiological evaluation in view of orthopedic surgery. During the preoperative examination, echocardiography was performed, which showed multiple flows in the interventricular septum as well as a diastolic flow at the level of the pulmonary valve. Due to these findings, it was decided to perform a cardiac catheterization. On cardiac catheterization, it was diagnosed an anatomical variation of the circumflex branch of the left coronary artery arising from the main stem of the pulmonary artery. Significant stenosis was remarkable, as well as collateral circulation of both the circumflex and the left anterior descending artery with the right coronary artery. The child finally at the age of 11 underwent cardiothoracic surgery. To conclude, during asymptomatic cardiological evaluation, we should always think about the possibility of anatomic variations of the coronary arteries. Missing these types of anomalies may predispose to inadvertent life-threatening complications or sudden death.

## Introduction

Coronary anatomy principally consists of the left main and right coronary artery (LMCA, RCA). These arteries stem respectively from the left and right Valsalva sinuses. The LMCA afterwards provides the left anterior descending (LAD) and the left circumflex (LCX) coronary artery. Furthermore, the above mentioned arteries divide into smaller branches which then penetrate the epicardium and provide blood supply to the transmural myocardium [1].

In contrast to normal anatomy, congenital coronary anatomical variations range from 0.2 to 1.3% upon various either angiographic or cadaveric series [2,3].

According to the literature, the most common coronary anatomical variation is the anomalous left circumflex artery (ALCx) that its incidence is approximately 0.37 to 0.7% [4,5]. In most of the cases, this anomaly may stem from a discrete ostium in the right sinus, or rarely as a central branch of the right coronary artery (RCA).

According to Shriki et al., coronary artery anomalies have been classified into haemodynamically significant, which may be correlated with sudden death, shunting or ischaemia and not haemodynamically significant anomalies similar to our case [6]. Of the reported cases with hemodynamic significance, the anatomical variation from the main branch of pulmonary artery may be diagnosed as myocardial infarction or left ventricular failure due to remarkable shunt immediately in the postpartum period. The anatomical variation of the left coronary artery arising from the main branch of pulmonary artery (ALCAPA) is also described in the medical literature as "Bland-Garland-White" syndrome. The incidence of the syndrome is reported in 0.25%-0.5% of congenital heart disease [1].

Treatment of the syndrome might be pharmaceutical or surgical (recommended even in asymptomatic patients) and it consists of reimplantation in the aorta or intrapulmonary baffling or grafting [1].

## Case presentation

An asymptomatic 9-year-old male had a past medical history of interventricular communication due to interventricular septal defect in infancy, which spontaneously closed without residual regurgitation. The child was referred for preoperative cardiac evaluation due to auscultated systolic murmur in view of orthopedic surgery (removal of a foreign body on the left foot). It is of paramount importance to point out, that the absence of a remarkable cardiac murmur does not exclude any severe cardiac disease. His routine biochemical parameters were within normal rates.

In the electrocardiogram (ECG) of 12 leads of the child at rest, there were observed negative T in the V1-V3 leads, which is a normal variant of the electrocardiogram in children and young women. It was also observed biphasic T in V4 which is sometimes present in the normal pediatric ECG.

Echocardiography revealed normal left ventricular contractility, with ejection fraction (EF) >65%. The internal dimensions of the left abdomen were increased with age. Its end-diastolic diameter (LVIDd) was 44 mm with normal values for the age and body weight of the child up to 41 mm. The thickness of the left ventricular wall at dilation was normal (intraventricular septum [IVSd] 7 mm). The left atrium showed mild dilation.

**Figure 1:**
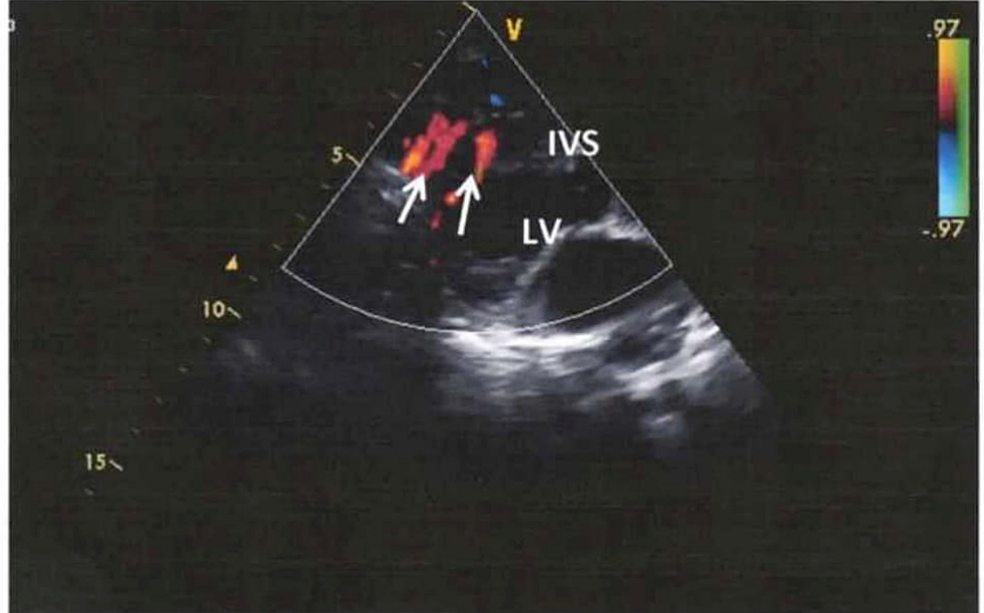
Echocardiogram (longitudinal axis). Colored flows (white arrows) are observed in the area of the interventricular septum (IVS), that correspond to collateral vessels.

The echocardiography also showed ventricular communication that has been closed in infancy and insignificant leak through it. Also, multiple pathological flows emerged in the area of the interventricular septum that gave the impression of multiple remaining muscular type of interventricular communications (Figure 1). Inside the myocardium of the left ventricle, elongated flows were detected which were also considered as potential communications.

In addition, the pulmonary artery stem developed a diastolic flow which, as it turned out, was due to an anomalous outflow of the left circumflex coronary arterial branch from the pulmonary artery. Due to the above findings in the echocardiography examination it was decided to perform cardiac catheterization and coronary angiography.

**Figure 2:**
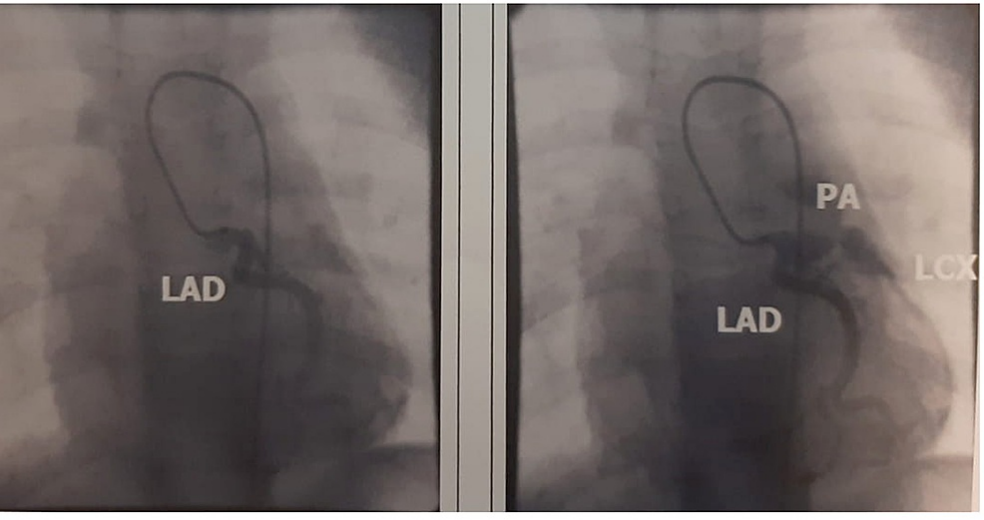
Selective coronary angiography of the left coronary artery: Lateral circulation imaging of the abnormal origin of the left circumflex coronary artery (LCX) from the pulmonary artery (PA) following selective left anterior descending coronary artery (LAD) catheterization that is depicted normal course.

Catheterization of the right heart chambers from the right femoral vein was performed percutaneously. Retrograde catheterization of the aorta, coronary arteries and left ventricle from the right femoral artery was then performed percutaneously (Figure 2).

During oximetry that was performed in the catheterization, the arterial blood saturation was normal. The ratio of pulmonary blood flow (Qp) to systemic blood flow (Qs) was calculated according to Fick principle (Qp / Qs = 1), which indicated no significant left-right blood shunt.

**Figure 3:**
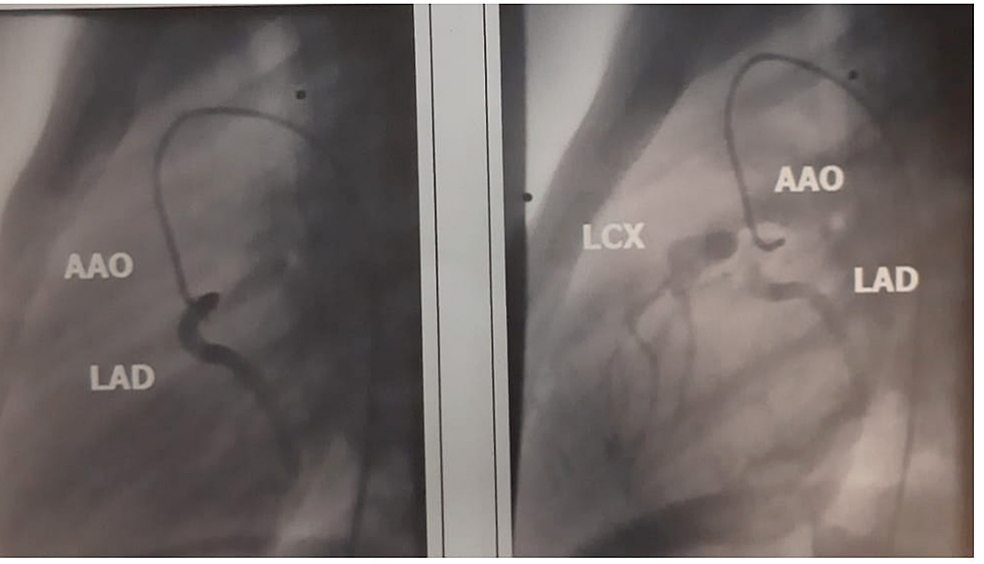
Left anterior descending coronary artery, branch of the left coronary artery (LAD) is normally imaged. The left circumflex coronary artery (LCX) and its abnormal outflow from the retrograde pulmonary artery (PA) are then imaged through an extensive collateral circulation. AAO: Ascending aorta

Normal outflow of the left anterior descending artery (LAD) from the left atrium of the Valsalva and abnormal outflow of the left circumflex coronary artery (LCX) from the pulmonary artery (PA) (anomalous left circumflex coronary artery from pulmonary artery [ALCAPA]) was detected after injection of the contrast agent into the protrusion of the LAD (Figure 3).

**Figure 4:**
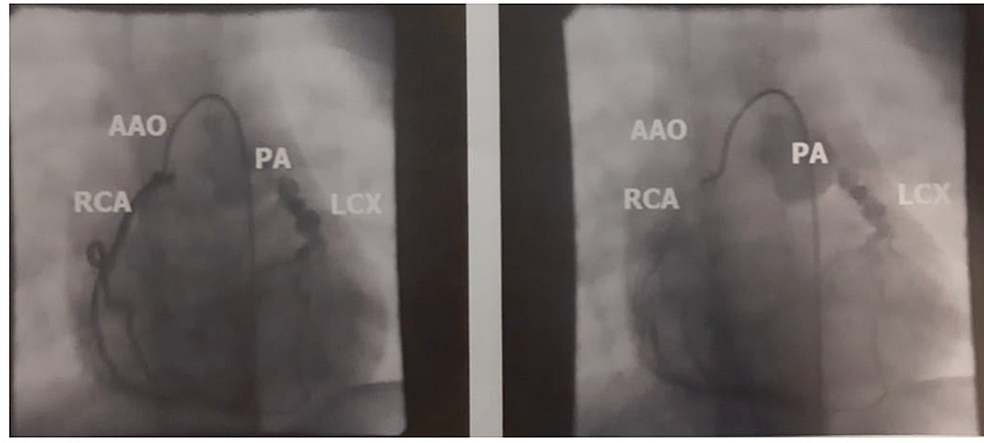
Selective right coronary artery (RCA) coronary angiography. RCA is the predominant coronary artery as the posterior descending branch emerges from it. We observe that there is an extensive collateral circulation network between the right coronary artery and the left circumflex coronary artery (LCX) which is outlined retrospectively. The pulmonary artery (PA) from which LCX emerges is then depicted retrospectively. AAO: Ascending aorta

Right coronary angiography showed a predominant right coronary artery (RCA). There was a retrospective imaging of the left parietal and pulmonary artery through an extensive lateral coronary network between the RCA and the LCX (Figure 4).

RCA is the predominant coronary artery as the posterior descending branch emerges from it. We observed also that there was an extensive collateral circulation network between the right coronary artery and the LCX which is outlined retrospectively by this rich blood supply. The pulmonary artery from which LCX emerges was then outlined retrospectively.

Myocardial stress echo and nuclear scintigraphy were finally performed. A myocardial stress echo test revealed asymptomatic ischemia with a decrease in the ST interval in leads II, III, aVF, V4-V6 indicating possible development of ischemia in the lower and lateral wall of the left ventricle.

The child was operated at the age of 11, two years after the initial diagnosis, due to parental refusal to consent to the risks of an open-heart surgery, in terms of mortality and morbidity. Finally, the child underwent successful reimplantation of LCX in the aorta. It is remarkable and it is extremely rare for an asymptomatic child to find an abnormal outflow of circulating LCX from the pulmonary artery.

## Discussion

Coronary artery anomalies can be attributed to embryological events during the third week of fetal development [7]. Failure of the normal connection of the left coronary artery bud to the aorta results in an abnormal connection to the pulmonary artery. The abnormal origin can be situated in the main pulmonary artery or proximal branches. In utero, with equal pulmonary and aortic pressures, satisfactory perfusion of the ALCAPA can occur. After birth, the pulmonary artery pressure fails and left coronary artery perfusion decreases.

Sudden cardiac death, ischemia or shunting are hemodynamically significant clinical manifestations of patients with coronary artery anomalies. There may be the result from contortion of the vessel’s slit-like, tangential origin that during excessive exercise may lead to ischemia and lethal arrhythmias [8].

The anatomical variation of the LCX from the pulmonary artery, usually is diagnosed in childhood and it is correlated with other clinically important congenital heart diseases, such as pulmonary valve stenosis, aortic coarctation, subaortic stenosis and cardiac defects (patent ductus arteriosus) [8,9]. In most cases, left circumflex coronary artery originating from the right sinus of Valsalva is well described and it has no clinical interest. Early diagnosis with angiographic evidence of the variations of the coronary arteries anatomy, is of paramount importance in patients who are preoperatively evaluated to undergo coronary artery surgery or cardiac valve replacement. Failure to demonstrate coronary artery anatomical variations may lead to hazardous interpretations of coronary artery anatomy and might be lethal for patients [9]. The gold standard for diagnosis is coronary angiography, similarly to our case, that allows optimal demonstration of potential collateral vessels and the degree of shunt.

Treatment of the syndrome might be pharmaceutical or surgical. Surgical management may include ligation of the LCX at its origin alone, reimplantation of the LCX into the aorta, a coronary artery bypass graft (CABG) or even a transpulmonary artery aortocoronary anastomosis [10-12].

## Conclusions

Coronary arteries variant anatomy represents variability in clinical image of patients and their prognosis depends highly on the course and their relation to the great vessels. Presumably, early detection of coronary arteries anomalies is crucial. The abnormal emanation of the left circumflex coronary arterial branch from the main stem of the pulmonary artery is considered a rare variation. Although it is usually asymptomatic, the clinical significance of this anatomical variation is apparent from its association with sudden cardiac death, syncope and arrhythmias as complications of myocardial ischemia.
